# Supplementary material for: First virological and pathological study of Göttingen Minipigs with Dippity Pig Syndrome (DPS)
Source: PLoS One. 2023 Jun 15;18(6):e0281521. doi: 10.1371/journal.pone.0281521 (PMC10270609; doi:10.1371/journal.pone.0281521)
Supplement: S1 Table — (DOCX) [file pone.0281521.s001.docx]

**Supplementary Table 1.** Data on sex, age, housing and other parameters concerning the studied GöMP with and without DPS

| **Animal*** | | **Age**  **(months)** | **Sex** | **Housing**  **(group/individual)** | **Vaccination** | **Status of the animal**  **(Colony, pre-study, study, post-study)** | **Littermates** | **Exposure to sunlight** | **Treatment** | **Outcome** |
| --- | --- | --- | --- | --- | --- | --- | --- | --- | --- | --- |
| 1 | 901 | 12 | Male | Individual | No | Colony | No | No | None | Euthanized |
| 2 | 239185 | 4-5 | Female | Group | No | Study (untreated control) | No | No | NSAID** | Recovered |
| 3 | 237587 | 15 | Female | Individual | No | Colony | No | No | None | Euthanized |
| 4 | 343528 (unaffected) | Not registered | Male | Individual | No | Colony | No | No | None | Not assessed |
| 5 | 349753 | 6 | Female | Group | No | Colony | No | No | None | Euthanized |
| 6 | 342036 | 4 | Female | Group | No | Colony | No | No | None | Euthanized |
| 7 | 342746 | 4 | Male | Group | No | Colony | No | No | NSAID | Recovered |
| 8 | 343061 | 5 | Male | Individual | No | Study | No | No | NSAID | Recovered |
| 9 | 314 | 2 | Female | Group | No | Colony | No | No | NSAID | Recovered |

*Short number used in this manuscript and numbers used by the breeder; ** non-steroidal anti-inflammatory drug
