# Supplementary material for: First virological and pathological study of Göttingen Minipigs with Dippity Pig Syndrome (DPS)
Source: PLoS One. 2023 Jun 15;18(6):e0281521. doi: 10.1371/journal.pone.0281521 (PMC10270609; doi:10.1371/journal.pone.0281521)
Supplement: S2 Table — (DOCX) [file pone.0281521.s002.docx]

**Supplementary Table 2.** Clinical signs, macroscopic and histopathological/microscopic findings of the analyzed pigs.

| **Animal*** | | **Clinical signs** | **Macroscopic findings** | **Histopathological/Microscopic findings** |
| --- | --- | --- | --- | --- |
| 1 | 901 | Acute lesions on dorsal spine, lesions wet with exudate, parallel lesions, arching of back, increased activity | Exudative lesion | Acute lesions, characterized by epidermal necrosis, epidermal intercellular edema (spongiosis), hemorrhage/congestion of blood vessels in the papillary dermis, subepidermal vesicle formation and low-grade infiltrations of inflammatory cells (dominated by granulocytes) |
| 2 | 239185 | Acute lesions on dorsal spine, lesions wet with exudate, vocalization | Exudative lesion | Acute lesions, including epidermal necrosis, subepidermal edema, subepidermal infiltration of neutrophilic granulocytes and vesicle formation |
| 3 | 237587 | Acute lesions on dorsal spine, lesions wet with exudate, arching of back, vocalization, sensitivity to touch | Exudative lesion | Acute lesions, including epidermal necrosis, epidermal / subepidermal edema, subepidermal infiltration of inflammatory cells (neutrophilic granulocytes) and vesicle formations |
| 4 | 343528 (unaffected animal) | None | Necropsy not performed | Histopathology not performed |
| 5 | 349753 | Acute lesions on dorsal spine, lesions wet with exudate, parallel lesions, arching of back, vocalization, sensitivity to touch | Exudative lesion | Acute lesions, including crust, epidermal necrosis, epidermal edema, subepidermal infiltration of mixed inflammatory cells. |
| 6 | 342036 | Acute lesions on dorsal spine, lesions red and wet with exudate, two parallel lesions, sensitivity to touch, arching of back | Exudative red lesion | Acute lesions characterized by epidermal necrosis, epidermal intercellular edema (spongiosis), subepidermal infiltration of mixed inflammatory cells (dominated by granulocytes), hemorrhage/congestion of blood vessels in papillary dermis and subepidermal/dermal vesicle formation |
| 7 | 342746 | Acute lesions on dorsal spine, lesions wet with exudate, parallel lesions, arching of back, sensitivity to touch, evidence of healing by day 2 after onset of symptoms. Sampling done 16 days after onset of symptoms. | Exudative red lesion | Parakeratotic hyperkeratosis, consistent with healing of the lesions. |
| 8 | 343061 | Acute lesions on dorsal spine, lesions wet with exudate, parallel lesions, unsteady gait | Exudative red lesion | Histopathology not performed |
| 9 | 314 | Acute lesions on dorsal spine, lesions wet with exudate, arching of back, sensitivity to touch | Exudative lesion | Acute lesions, including crust, epidermal necrosis, epidermal edema (spongiosis), subepidermal hemorrhage and infiltration of mixed inflammatory cells (dominated by granulocytes) occasionally forming intra-epidermal pustules, and vesicle formation. |

* Short number used in this manuscript and numbers used by the breeder
