# Supplementary material for: First virological and pathological study of Göttingen Minipigs with Dippity Pig Syndrome (DPS)
Source: PLoS One. 2023 Jun 15;18(6):e0281521. doi: 10.1371/journal.pone.0281521 (PMC10270609; doi:10.1371/journal.pone.0281521)
Supplement: S3 Table — (DOCX) [file pone.0281521.s003.docx]

**Supplementary Table 3.** Overview of the PCR results of six Göttinger Minipigs with dippity pig syndrome (DPS) and one not showing clinical signs.

|  | **PCMV** | **PCV1** | **PCV2** | **PCV3** | **PCV4** | **PLHV-1** | **PLHV-2** | **PLHV-3** | **PPV1** | **TTSuV1** | **TTSuV2** | **SARS-CoV-2** | **HEV** |  |
| --- | --- | --- | --- | --- | --- | --- | --- | --- | --- | --- | --- | --- | --- | --- |
| **pig # 1** | | | | | | | | | | | | | | |
| blood | - | - | - | 36.2 | - | - | - | - | - | - | - | - | - |  |
| liver | 26.8 | 26.9 | - | - | - | - | - | - | - | - | - | - | - |  |
| spleen | 27.9 | 35.4 | - | - | - | - | - | - | - | - | - | - | - |  |
| stomach* | - | - | - | - | - | - | - |  | - | - | - |  | - |  |
| sternum* | - | - | - | - | - | - | - |  | - | - | - |  | - |  |
| skin A* | 33.9 | - | - | - | - | - | - |  | - | - | - |  | - |  |
| skin B* | 35.1 | - | - | - | - | - | - |  | - | - | - |  | - |  |
| skin C* | 37.6 | - | - | - | - | - | - |  | - | - | - |  | - |  |
| **pig # 2** | | | | | | | | | | | | | | |
| PBMCs | - | 30.5 | - | - | - | - | - | - | - | - | - |  | - |  |
| skin A* | - | 37.3 | - | - | - | - | - |  | - | - | - |  | - |  |
| skin B* | - | 37.7 | - | 36.9 | - | - | - |  | - | - | - |  | - |  |
| skin C* | 38.15 | - | - | - | - | - | - |  | - | - | - |  | - |  |
| **pig # 3** | | | | | | | | | | | | | | |
| PBMCs | - | 34.5 | - | - | - | - | - | - | - | - | - |  | - |  |
| liver | 36.9 | - | - | - | - | - | - | - | - | - | - |  | - |  |
| spleen | - | - | - | - | - | - | - | - | - | - | - |  | - |  |
| skin A | - | - | - | - | - | - | - | - | - | - | - |  | - |  |
| skin B | - | - | - | - | - | - | - | - | - | - | - |  | - |  |
| skin C | - | - | - | - | - | - | - | - | - | - | - |  | - |  |
| **pig # 4 (animal without clinical signs)** | | | | | | | | | | | | | |  |
| blood | - | 36.42 | - | 35.36 | - | - | - | - | - | - | - |  |  |  |
| spleen |  |  |  |  |  |  |  |  | - | - | - | - | - |  |
| **pig # 6** | | | | | | | | | | | | | | |
| whole blood | negative | negative | negative | negative |  | negative | negative | negative |  |  |  |  |  |  |
| Plasma | negative | negative | negative | negative |  | negative | negative | negative |  |  |  |  |  |  |
| PBMCs | negative | negative | negative | negative |  | negative | negative | negative |  |  |  |  |  |  |
| liver | negative |  |  |  |  | negative | negative | negative |  |  |  |  |  |  |
| spleen | negative |  |  |  |  | negative | negative | negative |  |  |  |  |  |  |
| skin A | negative |  |  |  |  | negative | negative | negative |  |  |  |  |  |  |
| skin C | negative |  |  |  |  | negative | negative | negative |  |  |  |  |  |  |
| **pig # 7** | | | | | | | | | | | | | |  |
| serum | negative | negative | negative | negative |  | negative | negative | negative |  |  |  |  | negative |  |
| liver | negative | negative | negative | negative |  | negative | negative | negative |  |  |  |  | negative |  |
| spleen | negative | negative | negative | negative |  | negative | negative | negative |  |  |  |  | negative |  |
| skin A | negative | negative | negative | negative |  | negative | negative | negative |  |  |  |  | negative |  |
| skin C | negative | negative | negative | negative |  | negative | negative | negative |  |  |  |  | negative |  |
| **pig # 8** | | | | | | | | | | | | | |  |
| serum | negative | negative | negative | negative |  | negative | negative | negative | negative |  |  |  | negative |  |
| plasma | negative | negative | negative | negative |  | negative | negative | negative | negative |  |  |  | negative |  |

* FFPE material; numerical values = value of the cycle quantification; minus = negative result by qPCR and RT-qPCR, respectively; negative = negative result by conventional PCR, grey boxes = not tested
