# Supplementary material for: First virological and pathological study of Göttingen Minipigs with Dippity Pig Syndrome (DPS)
Source: PLoS One. 2023 Jun 15;18(6):e0281521. doi: 10.1371/journal.pone.0281521 (PMC10270609; doi:10.1371/journal.pone.0281521)
Supplement: S4 Table — (DOCX) [file pone.0281521.s004.docx]

**Supplementary Table 4** Detection of integrated PERVs in six Göttingen Minipigs with DPS and one minipig not showing clinical signs.

|  | **Molecular detection** | |
| --- | --- | --- |
|  | **PERV-C** | **PERV-A/C** |
| **pig # 1** | | |
| blood | 19.3 | - |
| liver | 21.8 | - |
| spleen | 15.8 | - |
| stomach* | 17.4 | - |
| sternum* | 21.4 | - |
| skin A* | 25.9 | - |
| skin B* | 22.1 | - |
| skin C* | 22.4 | - |
| **pig # 2** | | |
| PBMCs | 21.0 | - |
| skin A* | 22.5 | - |
| skin B* | 22.9 | - |
| skin C* | 21.2 | - |
| **pig # 3** | | |
| PBMCs |  |  |
| liver | 18.0 | - |
| spleen |  |  |
| skin A* |  |  |
| skin B* |  |  |
| skin C* |  |  |
| **pig # 4** (animal without clinical signs) | | |
| blood | 18.0 | - |
| spleen |  |  |
| **pig # 6** | | |
| whole blood | - | - |
| plasma | - | - |
| PBMCs | + | + |
| liver |  | + |
| spleen |  | + |
| skin A |  | + |
| skin C |  | + |
| **pig # 7** | | |
| serum | + | - |
| liver | + | - |
| spleen | + | - |
| skin A | + | - |
| skin C | + | - |
| **pig # 8** | | |
| serum | + | - |
| plasma | + | - |

numerical values = value of the cycle quantification; minus (-) = negative result by qPCR and conventional PCR, respectively; plus (+) = positive results by conventional PCR; grey boxes = not tested
