# Supplementary material for: First virological and pathological study of Göttingen Minipigs with Dippity Pig Syndrome (DPS)
Source: PLoS One. 2023 Jun 15;18(6):e0281521. doi: 10.1371/journal.pone.0281521 (PMC10270609; doi:10.1371/journal.pone.0281521)
Supplement: S5 Table — The selected taxa refer to the least common denominator of the data revealed by NGS approach independently of the taxon. (DOCX) [file pone.0281521.s005.docx]

**Supplementary Table 5.** Abundance of microbiological taxa obtained by a NGS approach for skin A (affected skin region) from animal # 3. The selected taxa refer to the least common denominator of the data revealed by NGS approach independently of the taxon.

| **taxa of interest** | **abundance**  **(in %)** | **read count** | **K-mers** | **B-Score** | **fraction**  **rRNA** |
| --- | --- | --- | --- | --- | --- |
| **viruses** | | | | | |
| Picornavirales sp. | 0.24107 | 930 | 170 | 0.200 | 0.000 |
| Totiviridae | 0.23664 | 913 | 255 | 0.231 | 0.997 |
| Porcine endogenous retrovirus C | 0.20169 | 778 | 5268 | 0.000 | 0.000 |
| Medicago sativa alphapartitivirus 1 | 0.00183 | 7 | 612 | 0.000 | 0.000 |
| Alfalfa mosaic virus | 0.00078 | 3 | 505 | 1.000 | 0.000 |
|  |  |  |  |  |  |
| **bacteria** | | | | | |
| Carnobacteriaceae | 10.59616 | 40874 | 35246 | 0.478 | 0.737 |
| Trichococcus paludicola | 0.32668 | 1260 | 900 | 0.100 | 0.690 |
| Carnobacterium inhibens | 0.17589 | 678 | 446 | 0.176 | 0.773 |
| Acinetobacter | 3.50568 | 13523 | 44242 | 0.836 | 0.630 |
| Paenibacillus | 3.31710 | 12796 | 18094 | 0.840 | 0.791 |
| Psychrobacillus | 0.08052 | 311 | 1661 | 0.250 | 0.400 |
| Sphingobacterium faecium | 0.16466 | 635 | 13194 | 0.100 | 0.061 |
| Pedobacter | 0.10574 | 408 | 4558 | 0.457 | 0.397 |
| Lelliottia amnigena | 1.28149 | 4943 | 2750 | 1.000 | 0.000 |
| Epilithonimonas | 0.70029 | 1426 | 11597 | 0.149 | 0.014 |
| Chryseobacterium indoltheticum | 0.30776 | 1187 | 4777 | 1.000 | 0.000 |
| Kaistella | 0.03290 | 127 | 443 | 0.548 | 0.000 |
| Moheibacter sediminis | 0.02843 | 110 | 498 | 0.300 | 0.404 |
| Aerococcus viridans | 0.76067 | 2934 | 1726 | 0.200 | 0.093 |
| Aerococcus urinaeequi | 0.41563 | 1603 | 1034 | 0.400 | 0.000 |
| Facklamia | 0.06891 | 266 | 801 | 0.706 | 0.908 |
| Lactococcus raffinolactis | 0.82851 | 3196 | 1717 | 1.000 | 0.817 |
| Streptococcus | 0.17248 | 665 | 2272 | 0.333 | 0.953 |
| Rheinheimera | 1.00809 | 3889 | 5646 | 0.200 | 0.764 |
| Pseudoclavibacter | 0.42857 | 1653 | 1402 | 0.067 | 0.854 |
| Microbacterium | 0.17143 | 661 | 1507 | 0.923 | 0.856 |
| Duganella | 0.56711 | 2188 | 1260 | 0.333 | 0.197 |
| Flavobacterium | 0.35105 | 1354 | 7172 | 0.604 | 0.244 |
| Corynebacterium callunae | 0.34338 | 1325 | 256 | 0.900 | 0.840 |
| Comamonas | 0.23056 | 889 | 1258 | 0.862 | 0.540 |
| Stenotrophomonas | 0.29418 | 1135 | 1492 | 0.914 | 0.480 |
| Lactobacillus | 0.18864 | 728 | 2790 | 0.926 | 0.908 |
| Glutamicibacter | 0.20051 | 773 | 1110 | 1.000 | 1.000 |
| Erwinia | 0.16697 | 644 | 1007 | 0.292 | 0.448 |
| Staphylococcus | 0.06982 | 269 | 2734 | 0.750 | 0.836 |
| Jeotgalicoccus | 0.04878 | 188 | 718 | 0.182 | 0.961 |
| Paracoccus | 0.09288 | 358 | 725 | 0.833 | 0.969 |
| Lachnospiraceae | 0.10342 | 399 | 4882 | 0.435 | 0.581 |
| Peptostreptococcaceae | 0.09820 | 379 | 1779 | 0.719 | 0.995 |
| Ruminococcus | 0.04353 | 168 | 1071 | 0.393 | 0.893 |
| Clostridium | 0.09376 | 362 | 2188 | 0.600 | 0.969 |
| Nocardiaceae | 0.03838 | 148 | 1302 | 0.737 | 0.926 |
| Dyadobacter | 0.03297 | 127 | 1533 | 0.905 | 0.947 |
| Vibrio | 0.02584 | 100 | 224 | 0.065 | 0.000 |
| Pseudomonas | 0.02996 | 116 | 1967 | 0.731 | 0.321 |
| Caulobacteraceae | 0.02112 | 81 | 2095 | 0.632 | 0.912 |
|  |  |  |  |  |  |
| **other taxa** | | | | | |
| Rhizopus microsporus | 5.33430 | 20577 | 942 | 0.800 | 0.012 |
| Lichtheimia ramosa | 1.97649 | 7624 | 465 | 0.100 | 0.002 |
| Debaryomyces hansenii | 0.04058 | 157 | 448 | 0.800 | 0.089 |
| Saccharomycetaceae | 0.75351 | 2907 | 206 | 0.185 | 0.006 |
| Penicillium | 0.19418 | 749 | 2847 | 0.571 | 0.217 |
