# Supplementary material for: First virological and pathological study of Göttingen Minipigs with Dippity Pig Syndrome (DPS)
Source: PLoS One. 2023 Jun 15;18(6):e0281521. doi: 10.1371/journal.pone.0281521 (PMC10270609; doi:10.1371/journal.pone.0281521)
Supplement: S6 Table — The selected taxa refer to the least common denominator of the data revealed by NGS approach independently of the taxon. (DOCX) [file pone.0281521.s006.docx]

**Supplementary Table 6.** Abundance of microbiological taxa obtained by a NGS approach for skin B (affected skin region) from minipig # 3. The selected taxa refer to the least common denominator of the data revealed by NGS approach independently of the taxon.

| **taxa of interest** | **abundance**  **(in %)** | **read count** | **K-mers** | **B-Score** | **fraction**  **rRNA** |
| --- | --- | --- | --- | --- | --- |
| **viruses** | | | | | |
| Picornavirales sp. | 0.16790 | 937 | 151 | 0.200 | 0.000 |
| Porcine endogenous retrovirus C | 0.14270 | 796 | 5145 | 0.000 | 0.000 |
| Alfalfa mosaic virus | 0.00360 | 20 | 450 | 0.100 | 0.800 |
| Plasmopara viticola lesion associated tombus-like virus 1 | 0.00180 | 10 | 50 | 1.000 | 0.0000 |
| Porcine astrovirus 4 | 0.00108 | 6 | 71 | 0.500 | 0.0000 |
|  |  |  |  |  |  |
| **bacteria** | | | | | |
| Epilithonimonas | 9.10655 | 50797 | 89338 | 0.273 | 0.065 |
| Chryseobacterium indoltheticum | 0.03877 | 216 | 965 | 1.000 | 0.000 |
| Kaistella | 0.12115 | 676 | 1333 | 0.654 | 0.007 |
| Lactococcus raffinolactis | 5.77426 | 32209 | 16084 | 1.000 | 0.705 |
| Streptococcus | 0.87601 | 4886 | 4688 | 0.414 | 0.992 |
| Erwinia | 3.36402 | 18765 | 9828 | 0.526 | 0.015 |
| Trichococcus paludicola | 0.45148 | 2518 | 298 | 0.400 | 0.821 |
| Carnobacterium | 0.07712 | 430 | 586 | 0.200 | 0.980 |
| Flavobacterium viscosus | 0.18344 | 1023 | 1350 | 0.200 | 0.174 |
| Rheinheimera | 1.59897 | 8919 | 8849 | 0.372 | 0.795 |
| Bacillaceae | 1.50864 | 8415 | 2864 | 0.205 | 0.993 |
| Pseudoclavibacter | 0.49417 | 2757 | 1440 | 0.200 | 0.877 |
| Microbacterium | 0.18073 | 1008 | 1964 | 0.852 | 0.867 |
| Comamonas | 0.20787 | 1159 | 2407 | 0.660 | 0.316 |
| Variovorax | 0.05124 | 286 | 777 | 0.913 | 0.453 |
| Lactobacillus delbrueckii | 0.35377 | 1973 | 1103 | 1.000 | 0.988 |
| Weissella | 0.13241 | 739 | 1873 | 0.800 | 0.975 |
| Limosilactobacillus | 0.07928 | 442 | 1390 | 0.952 | 0.957 |
| Corynebacterium casei | 0.17583 | 981 | 1108 | 0.200 | 1.000 |
| Peptostreptococcaceae | 0.39591 | 2208 | 2415 | 0.729 | 0.996 |
| Sphingobacterium | 0.15459 | 862 | 10572 | 0.737 | 0.179 |
| Pedobacter heparinus | 0.02967 | 166 | 658 | 0.300 | 1.000 |
| Rothia | 0.16078 | 897 | 1554 | 0.824 | 0.957 |
| Glutamicibacter arilaitensis | 0.08914 | 497 | 704 | 0.100 | 1.000 |
| Acinetobacter venetianus | 0.07933 | 442 | 472 | 1.000 | 0.000 |
| Lachnospiraceae | 0.22600 | 1261 | 8056 | 0.512 | 0.745 |
| Staphylococcus | 0.20612 | 1150 | 3714 | 0.720 | 0.981 |
| Clostridium | 0.21311 | 1189 | 3093 | 0.762 | 0.989 |
| Stenotrophomonas | 0.17160 | 957 | 1564 | 0.808 | 0.407 |
| Enterobacteriaceae | 0.19609 | 1094 | 3154 | 0.738 | 0.273 |
| Ruminococcus champanellensis | 0.09098 | 507 | 596 | 0.545 | 1.000 |
| Rhodococcus | 0.13290 | 741 | 1605 | 0.722 | 0.876 |
| Sphingomonas | 0.11256 | 628 | 1932 | 0.667 | 0.979 |
| Aerococcus | 0.08776 | 490 | 1139 | 0.900 | 0.915 |
| Pseudomonas | 0.10034 | 560 | 5145 | 0.878 | 0.498 |
| Rhodobacteraceae | 0.08106 | 452 | 2688 | 0.661 | 0.901 |
| Shewanella | 0.06205 | 346 | 401 | 0.235 | 0.962 |
| Turicibacter sanguinis | 0.03973 | 222 | 377 | 0.800 | 1.000 |
| Dyadobacter fermentans | 0.03114 | 174 | 516 | 0.200 | 1.000 |
| Nocardioides | 0.03298 | 184 | 1647 | 0.636 | 0.965 |
| Bradyrhizobium | 0.03021 | 168 | 1367 | 1.000 | 0.044 |
| Brevundimonas | 0.02686 | 150 | 1440 | 0.765 | 0.938 |
| Bifidobacterium | 0.02579 | 144 | 2391 | 0.950 | 0.941 |
| Oxalobacteraceae | 0.02254 | 126 | 1099 | 0.414 | 0.633 |
| Rhizobiaceae | 0.02203 | 123 | 1758 | 0.412 | 0.672 |
| Hymenobacter | 0.02017 | 113 | 1195 | 0.917 | 1.000 |
| Prevotella | 0.01536 | 86 | 1588 | 0.348 | 0.769 |
| Rikenellaceae | 0.01589 | 89 | 478 | 0.059 | 1.000 |
| Chitinophagaceae | 0.01575 | 88 | 377 | 0.037 | 0.051 |
|  |  |  |  |  |  |
| **other taxa** | | | | | |
| Phycomyces blakesleeanus | 16.85101 | 93995 | 356 | 0.100 | 0.913 |
| Rhizopus microsporus | 3.94577 | 22010 | 972 | 0.900 | 0.007 |
| Lichtheimia ramosa | 1.43582 | 8009 | 474 | 0.100 | 0.003 |
| Debaryomyces hansenii | 0.46812 | 2611 | 10362 | 1.000 | 0.029 |
| Saccharomycetaceae | 0.40067 | 1352 | 265 | 0.158 | 0.010 |
| Trichosporonaceae | 0.05918 | 330 | 396 | 0.742 | 0.787 |
