# Supplementary figures and images for: First virological and pathological study of Göttingen Minipigs with Dippity Pig Syndrome (DPS)
Source: PLoS One. 2023 Jun 15;18(6):e0281521. doi: 10.1371/journal.pone.0281521 (PMC10270609; doi:10.1371/journal.pone.0281521)

## Slide 1
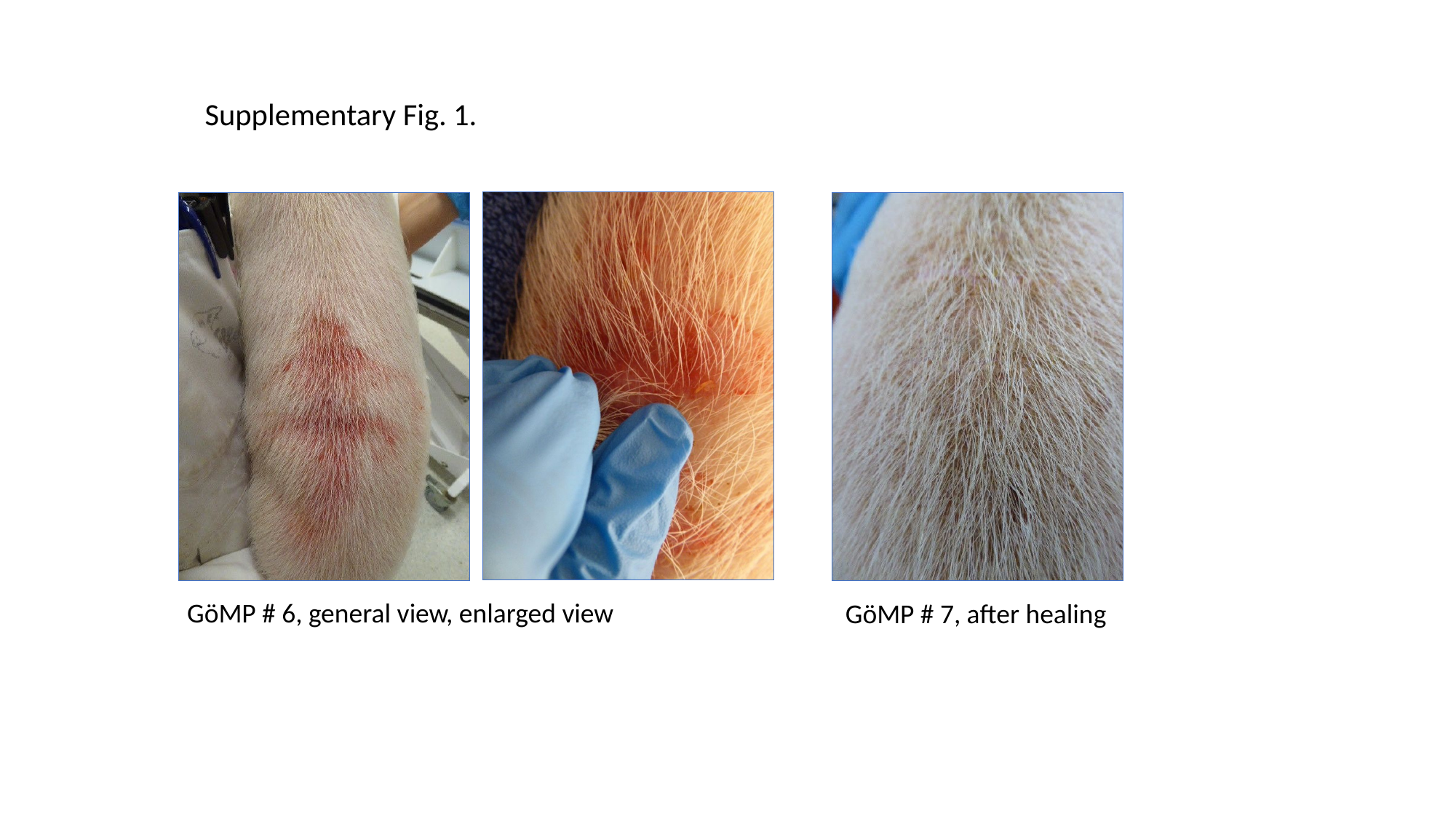

Supplementary Fig. 1.
GöMP # 6, general view, enlarged view
GöMP # 7, after healing

Supplement: S1 Fig — (PPTX) [file pone.0281521.s007.pptx]
